# Solid-phase total synthesis and structural confirmation of antimicrobial longicatenamide A

**DOI:** 10.3762/bjoc.18.166

**Published:** 2022-11-18

**Authors:** Takumi Matsumoto, Takefumi Kuranaga, Yuto Taniguchi, Weicheng Wang, Hideaki Kakeya

**Affiliations:** 1 Department of System Chemotherapy and Molecular Sciences, Division of Medicinal Frontier Sciences, Graduate School of Pharmaceutical Sciences, Kyoto University, Yoshida, Sakyo-ku, Kyoto 606-8501, Japan

**Keywords:** antimicrobial, longicatenamides, peptidic natural product, solid-phase synthesis, total synthesis

## Abstract

Longicatenamides A–D are cyclic hexapeptides isolated from the combined culture of *Streptomyces* sp. KUSC_F05 and *Tsukamurella pulmonis* TP-B0596. Because these peptides are not detected in the monoculture broth of the actinomycete, they are key tools for understanding chemical communication in the microbial world. Herein, we report the solid-phase total synthesis and structural confirmation of longicatenamide A. First, commercially unavailable building blocks were chemically synthesized with stereocontrol. Second, the peptide chain was elongated via Fmoc-based solid-phase peptide synthesis. Third, the peptide chain was cyclized in the solution phase, followed by simultaneous cleavage of all protecting groups to afford longicatenamide A. Chromatographic analysis corroborated the chemical structure of longicatenamide A. Furthermore, the antimicrobial activity of synthesized longicatenamide A was confirmed. The developed solid-phase synthesis is expected to facilitate the rapid synthesis of diverse synthetic analogues.

## Introduction

Naturally occurring bioactive compounds can serve as both drug leads and research tools for chemical biology [1]. Because the rediscovery rate of these compounds has increased in the last few decades, new approaches to explore natural products are in demand [2]. To this end, the combined-culture strategy has been applied to discover new natural products. For example, the mycolic acid-containing bacterium *Tsukamurella pulmonis* TP-B0596 can influence the biosynthesis of cryptic natural products [3]. Additionally, we have developed several highly sensitive labeling reagents to detect and identify scarce and cryptic natural products [4]. Integrating the combined-culture strategy and new labeling reagents has led to the detection and structural determination of several unprecedented secondary metabolites [5–7].

**Figure 1:**
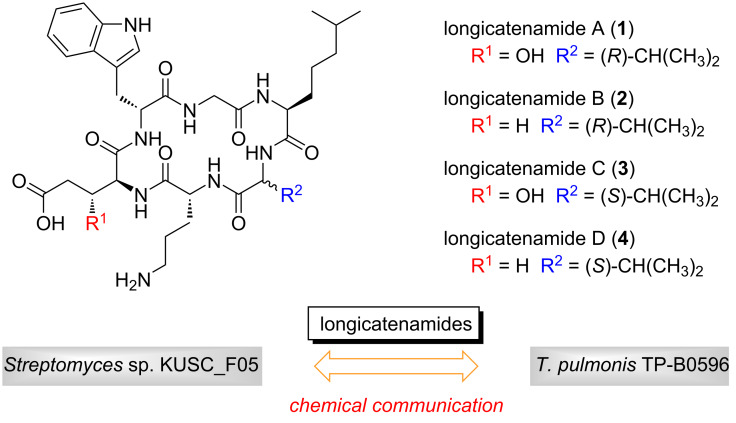
Structures of longicatenamides A–D (**1**–**4**).

Longicatenamides A–D (**1**–**4**, Figure 1) are cyclic hexapeptides isolated from the combined-culture of *Streptomyces* sp. KUSC_F05 and *T. pulmonis* TP-B0596 [8]. The planar structures were determined by analyzing two-dimensional (2D) nuclear magnetic resonance (NMR) spectra and mass spectrometry (MS) data, and the absolute configurations of their component amino acids were elucidated by using highly sensitive reagents that we recently developed [4]. Among the isolated longicatenamides, compound **1** exhibits weak but preferential antimicrobial activity against *Bacillus subtilis*. Because peptides **1**–**4** are not detected in the monoculture broth of *Streptomyces* sp. KUSC_F05, they are key tools for understanding chemical communication in the microbial world. To elucidate the role of compounds **1**–**4** in the microbial world, developing a strategy to synthesize compounds **1**–**4**, including future derivatization to produce probe molecules, is required. Herein, we report the total synthesis of peptide **1** by Fmoc-based solid-phase peptide synthesis [9] and the evaluation of its antimicrobial activity. The present study confirmed our proposed structure of **1**, which was determined by the use of our original labeling reagents [4].

## Results and Discussion

Although the solution-phase total synthesis of an analogue of longicatenamycin A has been reported [10], a solid-phase strategy can facilitate the production of a wider variety of analogous compounds than solution-phase synthesis [11]. Consequently, in this study, compound **1** was synthesized using a solid-phase strategy for future derivatization to produce probe molecules for deciphering microbial chemical communication.

**Scheme 1:**
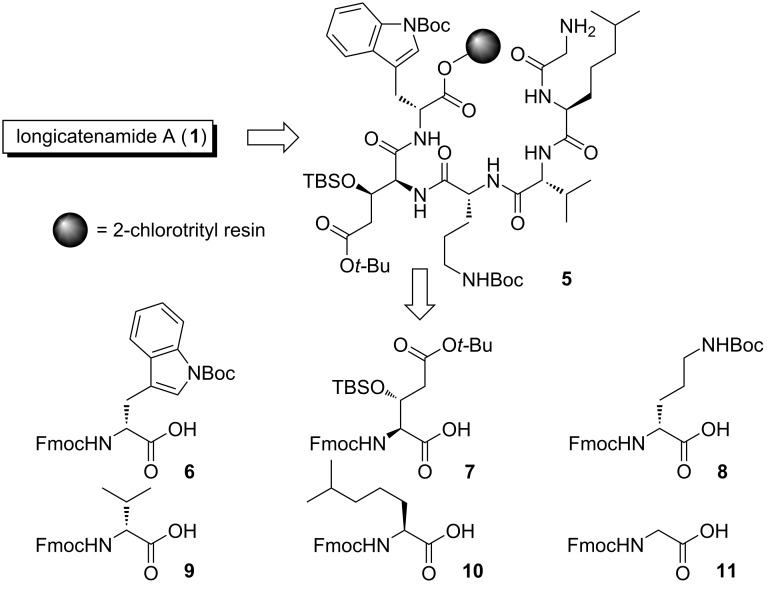
Retrosynthesis of longicatenamycin A (**1**).

The retrosynthesis of peptide **1** is displayed in Scheme 1. First, the cyclic peptide **1** was linearized by retrosynthesis, and acid-labile protecting groups were attached onto the reactive side chain. The biomimetic synthesis of cyclic peptides often enables efficient synthesis [12–13] and provides insights into the biosynthesis pathways of these peptides [14]. However, the biosynthetic gene clusters of compounds **1**–**4** remain unidentified. Therefore, the least sterically hindered amine, namely the amino group of glycine, was selected as a nucleophile of the cyclization reaction in this study. Second, to realize the solid-phase synthesis, the C-terminus of the linear peptide was connected to 2-chlorotrityl resin [15] to give resin-bound peptide **5**. Peptide **5** was divided into six building blocks **6**–**11** by retrosynthesis.

**Scheme 2:**
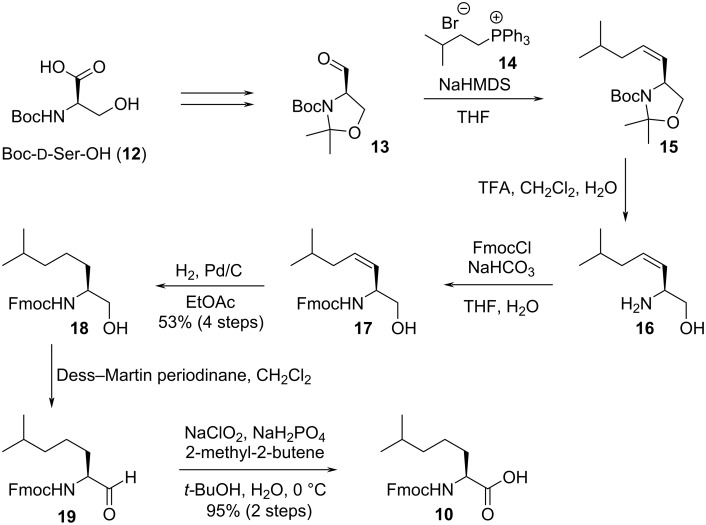
Synthesis of building block **10**.

At the beginning of the total synthesis, commercially unavailable building blocks **7** and **10** were chemically constructed from readily available starting materials. The synthesis of building block **10** commenced with the synthesis of compound **15** through Wittig reaction of Garner’s aldehyde (**13**) [16], which was readily obtained from *tert*-butyloxycarbonyl (Boc)-protected ᴅ-serine **12** (Scheme 2). Treatment of the olefin **15** with trifluoroacetic acid (TFA) cleaved the Boc protecting group and the acetonide to deliver unsaturated amino alcohol **16**. The amino group in **16** was protected by the fluorenylmethyloxycarbonyl (Fmoc) protecting group for solid-phase peptide synthesis, and then hydrogenation of the double bond in **17** provided intermediate **18**. Oxidation of the alcohol **18** to acid **10** was realized with the combination of Dess–Martin oxidation [17–18] and Pinnick oxidation [19].

**Scheme 3:**
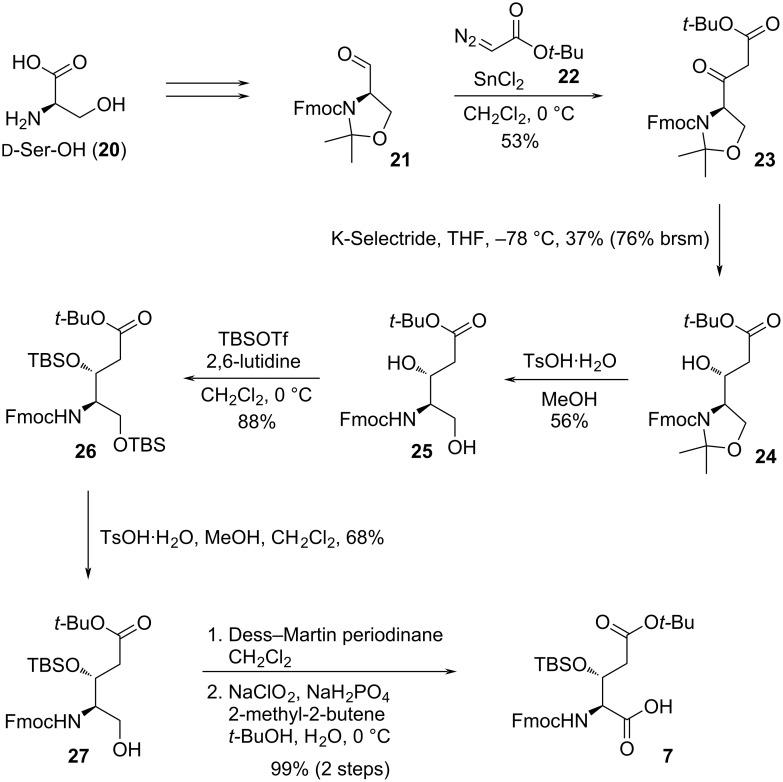
Synthesis of building block **7**.

Another unusual amino acid **7** was also synthesized from ᴅ-serine (**20**, Scheme 3). The SnCl_2_-catalyzed coupling reaction [20] between **21** and **22** afforded β-keto ester **23**, which was then reduced to the corresponding β-hydroxy ester **24** by K-Selectride (dr > 20:1), and subsequent acidic removal of the acetonide furnished diol **25**. The stereochemistry of the newly generated hydroxy group was determined using the modified Mosher’s method [21]. Protection of diol **25** by *tert*-butyl(dimethyl)silyl (TBS) group followed by selective deprotection of the primary alcohol led to **27**. Finally, acid **7** was obtained from alcohol **27** through the same two-step oxidation used to obtain compound **10**.

**Scheme 4:**
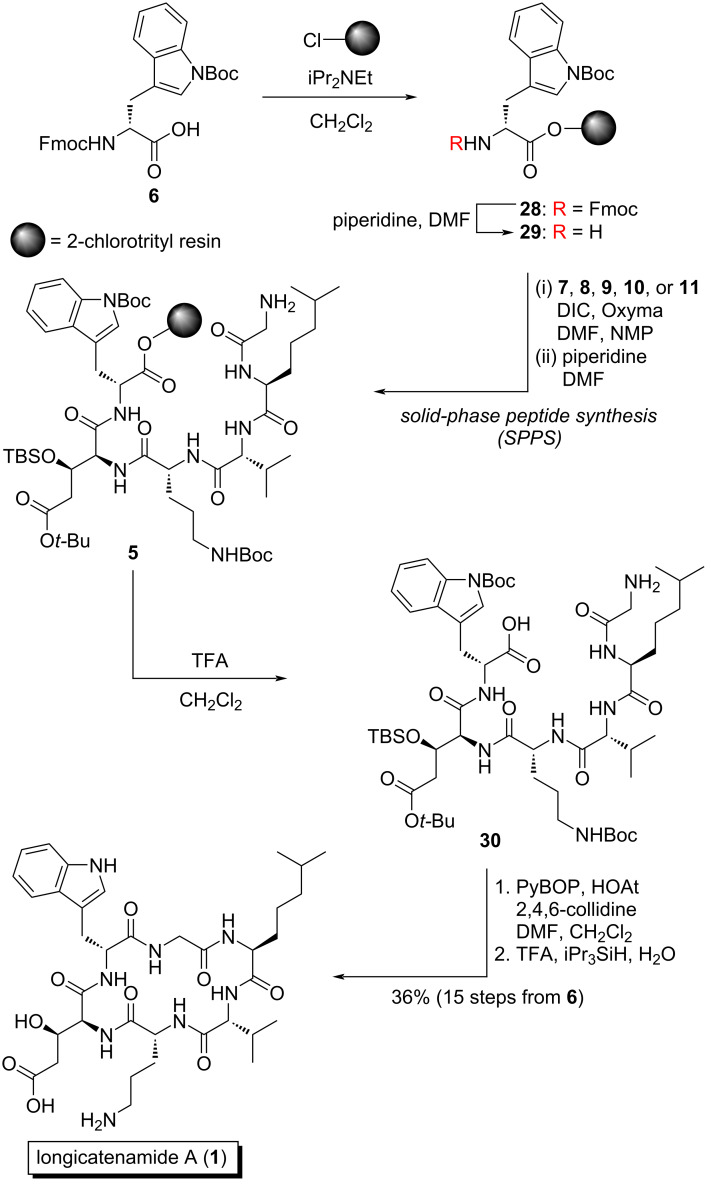
Total synthesis of longicatenamycin A (**1**).

Having synthesized the necessary building blocks, we turned our attention to construct resin-bound peptide **5** (Scheme 4). The assembly of this hexapeptide started with the loading of Fmoc-ᴅ-Trp(Boc)-OH (**6**) onto 2-chlorotrityl resin with iPr_2_NEt, which was followed by piperidine treatment to liberate resin-bound amine **29**. Then, five rounds of DIC/Oxyma-mediated amidation [22] and *N*^α^-deprotection with piperidine led to resin-bound peptide **5**. Treatment of **5** with TFA/CH_2_Cl_2_ 1:99 released **30** into the solution without unmasking the acid-labile protecting groups of the side chains. Subsequently, peptide **30** was cyclized by the action of PyBOP/HOAt [23–24] followed by treatment with TFA/iPr_3_SiH/H_2_O 95:2.5:2.5 to provide crude **1**. After reversed-phase high-performance liquid chromatography (HPLC) purification, longicatenamide A (**1**) was obtained with 36% yield over 15 steps starting from **6**.

**Figure 2:**
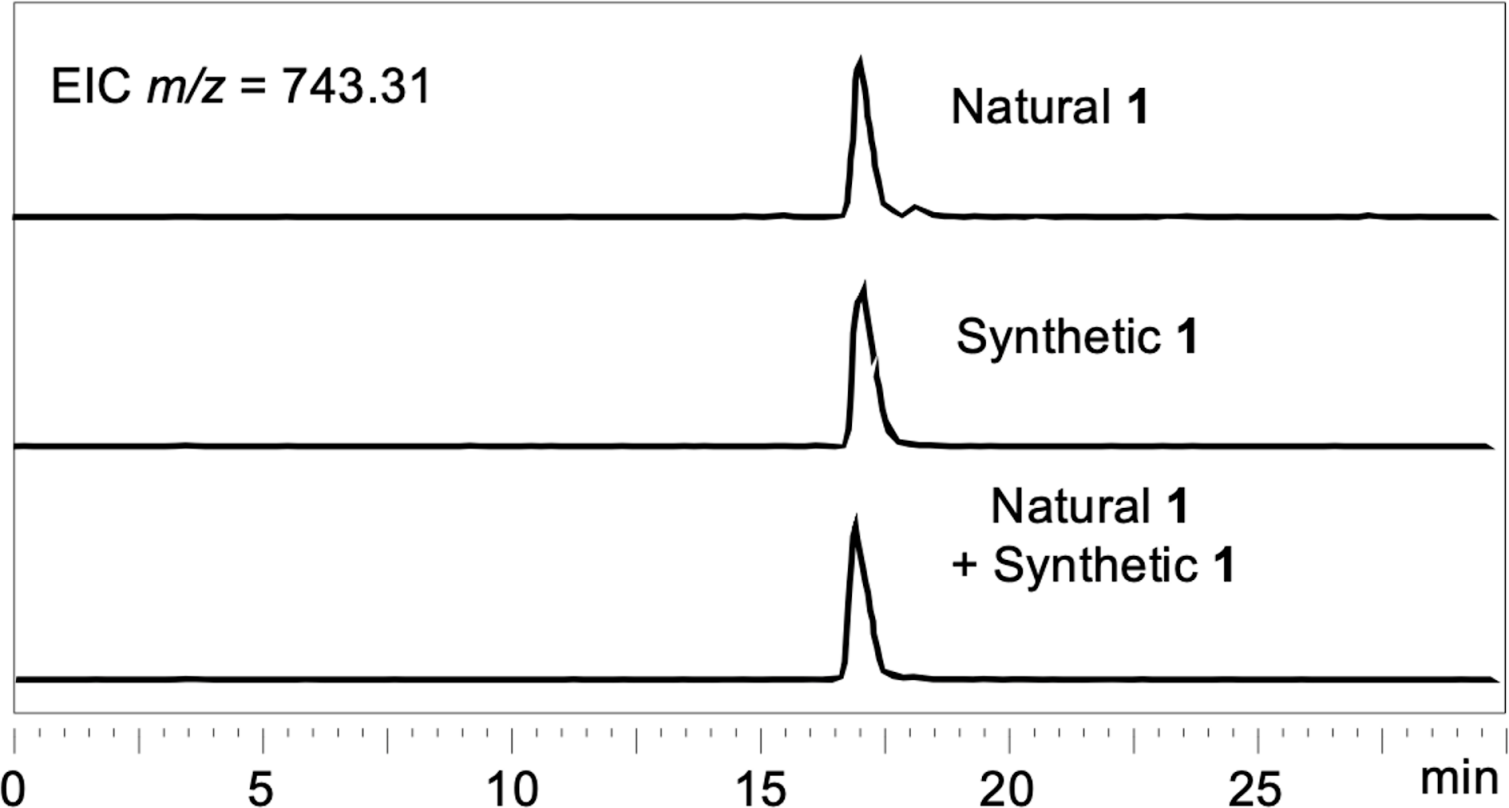
LC–MS extracted ion chromatograms (EICs) of synthesized and natural **1**. Column: Imtakt Cadenza CD-C18 3 × 150 mm; eluent: MeCN/H_2_O/TFA 30:70:0.05, isocratic, 0.2 mL/min; 40 °C.

The NMR spectra of synthesized compound **1** agreed with those of natural **1**. At this stage the confirmation of the identity of natural and synthesized compounds by structural determination using NMR spectroscopy is often difficult because the NMR spectra of peptidic products vary depending on the conditions in the NMR tube [25–29], such as concentration, pH, and purity. Thus, analyses of the NMR spectra of peptides sometimes lead to incorrect structural determination even though total synthesis of the proposed structures is successful [30]. In this study, synthesized and natural **1** were compared by collecting LC–MS data. As displayed in Figure 2, the retention time of synthesized **1** was identical to that of natural **1**. These results confirmed total synthesis of **1** and supported our proposed structure of isolated natural **1**.

Total synthesis sometimes invalidates the reported biological activity of the isolated natural product, which could be due to the presence of uncharacterized impurities. To verify the bioactivity of **1**, the antimicrobial activity of synthesized **1** was preliminary tested in this study following a previously reported method [8], revealing that chemically synthesized **1** (minimum inhibitory concentration (MIC) = 50 μM) exhibited moderate but selective antimicrobial activity against *Bacillus subtilis* similar to natural **1** (MIC = 100 μM).

## Conclusion

We accomplished solid-phase total synthesis of longicatenamide A (**1**). Initially, commercially unavailable building blocks **7** and **10** were chemically synthesized with stereocontrol. Then, the peptide chain was elongated by Fmoc-based solid-phase peptide synthesis. Finally, the cyclization of the peptide chain followed by simultaneous cleavage of all protecting groups in the solution phase afforded target compound **1**. The comparison of the chromatograms of synthesized and natural **1** corroborated the chemical structure of **1**. Further studies of the structure–activity relationship, identification of biosynthetic gene clusters, and detailed investigations of the combined-culture production of longicatenamides are currently underway in our laboratory.

## Supporting Information

File 1 Experimental procedures and compound characterization data.
