# Supplementary material for: Solid-phase total synthesis and structural confirmation of antimicrobial longicatenamide A
Source: Beilstein J Org Chem. 2022 Nov 18;18:1560–6. doi: 10.3762/bjoc.18.166 (PMC9679611; doi:10.3762/bjoc.18.166)
Supplement: File 1 — Experimental procedures and compound characterization data. [file Beilstein_J_Org_Chem-18-1560-s001.pdf]

BEILSTEIN JOURNAL OF ORGANIC CHEMISTRY

Supporting Information

for

Solid-phase total synthesis and structural confirmation of antimicrobial longicatenamide A

Takumi Matsumoto, Takefumi Kuranaga, Yuto Taniguchi, Weicheng Wang and Hideaki Kakeya

Beilstein J. Org. Chem. **2022,** *18,* 1560–1566. doi:10.3762/bjoc.18.166

Experimental procedures and compound characterization data

License and Terms: This is a supporting information file under the terms of the Creative Commons Attribution License (https://creativecommons.org/licenses/by/4.0). Please note that the reuse, redistribution and reproduction in particular requires that the author(s) and source are credited and that individual graphics may be subject to special legal provisions.
The license is subject to the *Beilstein Journal of Organic Chemistry* terms and conditions: (https://www.beilstein-journals.org/bjoc/terms)

Table of contents

General remarks S2

Synthesis and compound characterization S2–S7

NMR spectra of new compounds S8–S15

NMR spectra of **1** S15

HSQC and HMBC spectra of **23** (mixture of the rotamer) S16

General remarks

^{1}H and ^{13}C NMR spectra were recorded on a JEOL ECZ600 (600 MHz for ^{1}H NMR and 150 MHz for ^{13}C NMR) spectrometer. Chemical shifts are denoted in δ (ppm) relative to residual solvent peaks as internal standard ($CDCl_3$, ^{1}H δ 7.25, ^{13}C δ 77.2, DMSO-d_6, ^{1}H δ 2.50, ^{13}C δ 39.5). ESIMS and LC–MS experiments were recorded on a Shimadzu LCMS-IT-TOF. Optical rotations were recorded on a JASCO P-2200 polarimeter. High performance liquid chromatography (HPLC) experiments were performed with a SHIMADZU HPLC system equipped with an LC-20AD intelligent pump. All reactions sensitive to air and/or moisture were conducted under nitrogen atmosphere using dry, freshly distilled solvents, unless otherwise noted. All reagents were used as supplied unless otherwise stated. Analytical thin-layer chromatography (TLC) was performed using Merck silica gel 60 F_{254} pre-coated plates. Silica gel column chromatography was performed using 40–50 μm silica gel 60N (Kanto Chemical Co., Inc.).

Synthesis and compound characterization

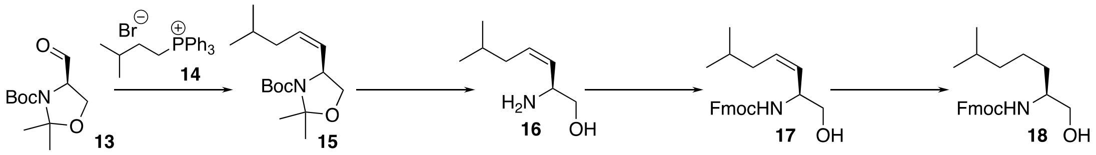

To a solution of compound **14** (2.66 g, 6.44 mmol) in THF (10 mL) was added NaHMDS (3.1 mL, 1.9 M in THF, 5.9 mmol) at 0 °C. After being stirred at 0 °C for 1 h, compound **13** (439 mg, 1.92 mmol) in THF (10 mL) was added dropwise to the solution. The reaction mixture was stirred at room temperature for 16 h, and then quenched with saturated aqueous NH_4Cl. The resulting solution was extracted with hexane/EtOAc 9:1 (three times). The combined organic layer was washed with brine, dried over $MgSO_4$, filtered, and concentrated in vacuo. The residue was roughly purified by silica gel column chromatography (hexane/EtOAc 97:3 to 90:10) to afford crude **15**, which was used in the next reaction without further purification.

To a solution of the above compound **15** in CH_2Cl_2 (13 mL) were added TFA (6 mL) and H_2O (1 mL) at room temperature. After being stirred for 1 h, the mixture was concentrated and azeotroped with toluene (three times) to afford crude **16**, which was used in the next reaction without further purification.

To a solution of the above compound **16** in THF/H_2O 2:1 (15 mL) were added $NaHCO_3$ (386 mg, 4.59 mmol) and FmocCl (594 mg, 2.30 mmol) at 0 °C. After being stirred at room temperature for 3 h, the reaction mixture was treated with saturated aqueous NH_4Cl. The resulting mixture was extracted with EtOAc (three times). The combined organic layer was washed with brine, dried over $MgSO_4$, filtered, and concentrated in vacuo. The residue was roughly purified by silica gel column chromatography (hexane/EtOAc 75:25 to 55:45) to afford crude **17**, which was used in the next reaction without further purification.

To a solution of the above compound **17** in EtOAc (15 mL) was added Pd/C (58.6 mg) and the mixture was stirred at room temperature under H_2 atmosphere for 5 h. The mixture was filtered through Celite and the filtrate

was concentrated in vacuo. The residue was purified by silica gel column chromatography (hexane/EtOAc 65:35) to afford of compound **18** (369 mg, 1.01 mmol, 53% for 4 steps) as a white powder: $[\alpha]_D^{20}$ −7.3 (*c* 0.25, MeOH); ^{1}H NMR (600 MHz, $CDCl_3$) δ 7.77 (d, 2H, *J* = 7.6 Hz), 7.60 (d, 2H, *J* = 7.6 Hz), 7.40 (t, 2H, *J* = 7.6 Hz), 7.32 (t, 2H, *J* = 6.9 Hz), 4.82 (d, 1H, *J* = 6.9 Hz), 4.43 (d, 2H, *J* = 6.9 Hz), 4.21 (t, 1H, *J* = 6.9 Hz), 3.68 (d, 2H, *J* = 9.0 Hz), 3.56 (m, 1H), 1.52 (m, 2H), 1.43 (m, 1H), 1.34 (m, 2H), 1.19 (m, 2H), 0.86 (d, 6H, *J* = 6.9 Hz); ^{13}C NMR (150 MHz, $CDCl_3$) δ 156.9. 144.0, 141.4, 127.8, 127.1, 125.1, 120.1, 66.7, 65.7, 53.4, 47.4, 38.8, 31.6, 27.9, 23.9, 22.7; HRMS (ESI) *m/z*: $[M+H]^+$ calcd for $C_{23}H_{30}NO_3^+$ 368.2220, found 368.2207.

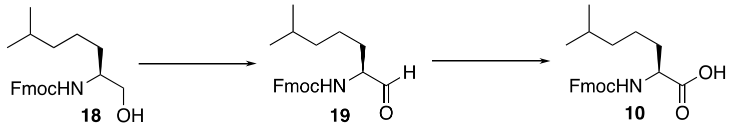

To a solution of compound **18** (227 mg, 0.617 mmol) in CH_2Cl_2 (7 mL) was added Dess–Martin periodinane (392 mg, 0.924 mmol) at room temperature. After being stirred at room temperature for 1.5 h, to the reaction mixture were added saturated aqueous solutions of $Na_2S_2O_3$ and $NaHCO_3$. The resulting mixture was extracted with EtOAc (three times). The combined organic layer was washed with brine, dried over $MgSO_4$, filtered, and concentrated in vacuo to afford crude **19**, which was used in the next reaction without further purification.

To a solution of the above compound **19** in *t*-BuOH (4.5 mL) and 2-methyl-2-butene (1.5mL) were added $NaClO_2$ (80%, 416 mg, 4.60 mmol) and $NaH_2PO_4 \cdot 2H_2O$ (372.6 mg, 2.39 mmol) in H_2O (2.25 mL) at 0 °C. After being stirred at 0 °C for 2 h, to the reaction mixture was added saturated aqueous NH_4Cl solution. The resulting mixture was extracted with EtOAc (three times). The combined organic layer was washed with saturated aqueous NH_4Cl, dried over $MgSO_4$, filtered, and concentrated in vacuo. The residue was purified by silica gel column chromatography (hexane/EtOAc 80:20 to 60:40) to afford compound **10** (cas: 329270-51-1, 222 mg, 0.583 mmol, 95% for 2 steps) as a white solid.

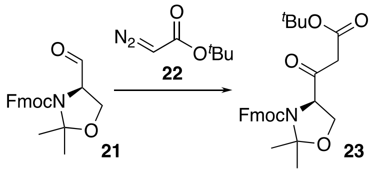

To a solution of compound **21** (2.07 g, 5.90 mmol) in CH_2Cl_2 (50 mL) were added **22** (3.35 g, 23.6 mmol) and $SnCl_2$ (565 mg, 2.98 mmol) at 0 °C. After being stirred at 0 °C for 15 h, to the reaction mixture were added 2% aqueous $KHSO_4$ and brine. The resulting mixture was extracted with $CHCl_3$ (five times). The combined organic layer was washed with brine, dried over $MgSO_4$, filtered, and concentrated in vacuo. The residue was purified by silica gel column chromatography (hexane/EtOAc 80:20 to 50:50) to afford compound **23** (1.45 g, 3.12 mmol, 53%) as a yellow oil: $[\alpha]_D^{20}$ +22 (*c* 1.0, MeOH); the compound gave complex NMR data due to the existence of multiple rotamers, therefore the structure was confirmed after conversion to **25**, ^{1}H NMR (600

MHz, $CDCl_3$) see Figure S3; ^{13}C NMR (150 MHz, $CDCl_3$) see Figure S4; HRMS (ESI) *m/z*: $[M+H]^+$ calcd for $C_{27}H_{32}NO_6^+$ 466.2224, found 466.2221.

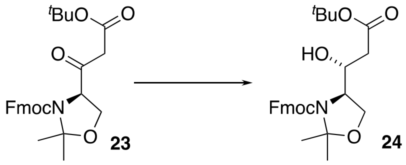

To a solution of compound **23** (2.37 g, 5.09 mmol) in THF (40 mL) was added K-Selectride (10.2 mL, 1 M in THF, 10.2 mmol) at −78 °C. After being stirred at −78 °C for 17 h, to the reaction mixture was added saturated aqueous NH_4Cl. The resulting mixture was extracted with EtOAc (three times). The combined organic layer was washed with brine, dried over $MgSO_4$, filtered, and concentrated in vacuo. The residue was purified by silica gel column chromatography (hexane/EtOAc 80:20 to 60:40) to afford **24** (882 mg, 1.89 mmol, 37%, brsm 76%) as a yellow oil: $[\alpha]_D^{20}$ +8.7 (*c* 1.0, MeOH); the compound gave complex NMR data due to the existence of multiple rotamers, therefore the structure was confirmed after conversion to **25**, ^1H NMR (600 MHz, $CDCl_3$) see Figure S5; ^{13}C NMR (150 MHz, $CDCl_3$) see Figure S6; HRMS (ESI) *m/z*: $[M+Na]^+$ calcd for $C_{27}H_{33}NO_6Na^+$ 490.2200, found 490.2195.

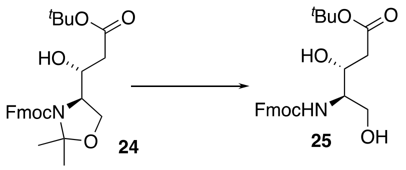

To a solution of compound **24** (917 mg, 1.96 mmol) in MeOH (30 mL) was added *p*-TsOH·H_2O (100 mg) at room temperature. After being stirred at room temperature for 9 h, to the reaction mixture was added saturated aqueous $NaHCO_3$. The mixture was extracted with EtOAc (three times). The combined organic layer was washed with brine, dried over $MgSO_4$, filtered, and concentrated in vacuo. The residue was purified by silica gel column chromatography (hexane/EtOAc 70:30 to 30:70) to afford **25** (465 mg, 1.09 mmol, 56%) as a yellowish oil: $[\alpha]_D^{20}$ +8.5 (*c* 0.16, MeOH); ^1H NMR (600 MHz, $CDCl_3$) δ 7.77 (d, 2H, *J* = 6.9 Hz), 7.61 (d, 2H, *J* = 6.9 Hz), 7.41(t, 2H, *J* = 7.6 Hz), 7.33(t, 2H, *J* = 7.6 Hz), 5.49(d, 1H, *J* = 9.6 Hz), 4.43 (d, 2H, *J* = 6.9 Hz), 4.33 (d, 1H, *J* = 10.3 Hz), 4.22 (t, 1H, *J* = 6.9 Hz), 3.89 (dd, 1H, *J* = 3.4 Hz, 11.0 Hz), 3.76 (dd, 1H, *J* = 4.8 Hz, 11.0 Hz), 3.66 (m, 1H), 2.49 (dd, 1H, *J* = 10.3 Hz, 16.9 Hz), 2.42 (dd, 1H, *J* = 2.7 Hz, 17.2 Hz), 1.46 (s, 9H); ^{13}C NMR (150 MHz, $CDCl_3$) δ 172.88, 156.84, 143.97, 141.43, 127.82, 127.17, 125.17, 120.11, 82.02, 69.93, 66.95, 65.00, 54.49, 47.36, 39.10, 28.17; HRMS (ESI) *m/z*: $[M+Na]^+$ calcd for $C_{24}H_{29}NO_6Na^+$ 450.1893, found 450.1892.

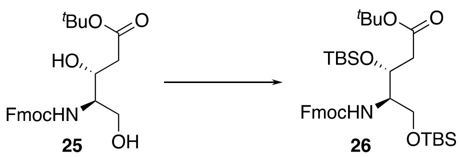

To a solution of compound **25** (663 mg, 1.55 mmol) in CH_2Cl_2 (15 mL) were added 2,6-lutidine (1.08 mL, 9.33 mmol) and TBSOTf (1.07 mL, 4.66 mmol) at 0 °C. After being stirred at 0 °C for 15 min, to the reaction mixture were added MeOH and saturated aqueous $NaHCO_3$. The resulting mixture was extracted with EtOAc (three times). The combined organic layer was washed with brine, dried over $MgSO_4$, filtered, and concentrated in vacuo. The residue was purified by silica gel column chromatography (hexane/EtOAc 95:5 to 90:10) to afford **26** (899 mg, 1.37 mmol, 88%) as a colorless oil: $[\alpha]_D^{20}$ +3.9 (*c* 1.0, MeOH); ^{1}H NMR (600 MHz, $CDCl_3$) δ 7.77 (d, 2H, *J* = 6.9 Hz), 7.60(d, 2H, *J* = 7.6 Hz), 7.40 (t, 2H, *J* = 6.9 Hz), 7.32 (t, 2H, *J* = 7.6 Hz), 5.03(d, 1H, *J* = 8.3 Hz), 4.47(t, 1H, *J* = 6.2 Hz), 4.37 (m, 2H), 4.26 (t, 1H, *J* = 6.9 Hz), 3.68 (m, 2H), 3.50 (t, 1H, *J* = 9.6 Hz), 2.45 (m, 2H), 1.44 (s, 9H), 0.91 (m, 18H), 0.09 (m, 12H); ^{13}C NMR (150 MHz, $CDCl_3$) δ 170.55, 156.41, 144.36, 141.65, 128.00, 127.36, 125.47, 120.31, 84.07, 67.12, 61.56, 55.94, 47.60, 41.52, 28.42, 26.26, 18.50, –4.17, –4.61, –4.98, –5.07; HRMS (ESI) *m/z*: $[M+H]^+$ calcd for $C_{36}H_{58}NO_6Si_2^+$ 656.3797, found 656.3796.

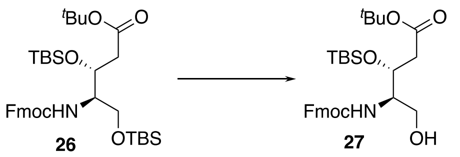

To a solution of **26** (158 mg, 0.241 mmol) in MeOH/CH_2Cl_2 (1:1, 6 mL) was added *p*-TsOH·H_2O (14.7 mg) at room temperature. After being stirred at room temperature for 80 min, the reaction mixture was added with Et_3N (100 μL). The mixture was concentrated in vacuo and the residue was purified by silica gel column chromatography (hexane/EtOAc = 80:20) to afford **27** (88.2 mg, 0.163 mmol, 68%) as a colorless oil: $[\alpha]_D^{20}$ +9.5 (*c* 1.00, MeOH); ^{1}H NMR (600 MHz, $CDCl_3$) δ 7.77 (d, 2H, *J* = 7.6 Hz), 7.60 (d, 2H, *J* = 7.6 Hz), 7.41 (t, 2H, *J* = 7.6 Hz), 7.32 (t, 2H, *J* = 6.9 Hz), 5.24 (d, 1H, *J* = 8.3 Hz), 4.40 (m, 3H), 4.25 (t, 1H, *J* = 6.2 Hz), 3.80 (br, 1H), 3.70 (br, 1H), 3.63 (br, 1H), 2.63 (s, 1H), 2.48 (m, 2H), 1.45 (br, 9H), 0.92 (br, 9H), 0.14 (br, 6H); ^{13}C NMR (150 MHz, $CDCl_3$) δ 170.54, 156.92, 143.98, 141.41, 127.81, 127.16, 125.19, 120.10, 81.24, 67.84, 67.00, 62.96, 56.24, 47.32, 40.79, 28.19, 25.88, 18.12, –4.39, –4.89; HRMS (ESI) *m/z*: $[M+H]^+$ calcd for $C_{30}H_{44}NO_6Si^+$ 542.2932, found 542.2933.

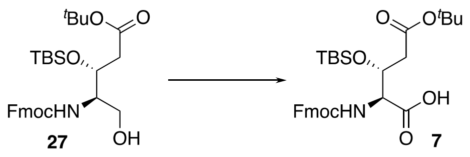

To a solution of compound **27** (454 mg, 0.838 mmol) in CH_2Cl_2 (10 mL) was added Dess–Martin

periodinane (535 mg, 1.26 mmol) at room temperature. After being stirred at room temperature for 1 h, to the reaction mixture were added saturated aqueous solutions of $Na_2S_2O_3$ and $NaHCO_3$. The resulting mixture was extracted with EtOAc (three times). The combined organic layer was washed with brine, dried over $MgSO_4$, filtered, and concentrated in vacuo to afford crude aldehyde, which was used in the next reaction without further purification.

To a solution of the above aldehyde in *t*-BuOH (6 mL) and 2-methyl-2-butene (2 mL) were added $NaClO_2$ (80%, 568 mg, 5.03 mmol) and $NaH_2PO_4 \cdot 2H_2O$ (471 mg, 3.02 mmol) in H_2O (3 mL) at 0 °C. After being stirred at 0 °C for 2 h, to the reaction mixture was added saturated aqueous NH_4Cl. The resulting mixture was extracted with EtOAc (three times). The combined organic layer was washed with saturated aqueous NH_4Cl, dried over $MgSO_4$, filtered, and concentrated in vacuo. The residue was purified by silica gel column chromatography ($CHCl_3$/MeOH 99:1) to afford **7** (460 mg, 0.828 mmol, 99%) as a colorless oil: $[\alpha]_D^{20}$ +6.6 (*c* 0.50, MeOH); ^1H NMR (600 MHz, $CDCl_3$) δ 7.77 (d, 2H, *J* = 7.6 Hz), 7.61 (t, 2H, *J* = 8.3 Hz), 7.40 (t, 2H, *J* = 6.9 Hz), 7.32 (t, 2H, *J* = 6.9 Hz), 5.50 (d, 1H, *J* = 8.9 Hz), 4.75 (t, 1H, *J* = 5.5 Hz), 4.54 (d, 1H, *J* = 8.3 Hz), 4.44 (m, 1H), 4.36 (m, 1H), 4.27 (t, 1H, *J* = 7.6 Hz), 2.55 (dd, 1H, *J* = 6.9 Hz, 16.5 Hz), 2.42 (dd, 1H, 6.2 Hz, 16.5 Hz), 1.45 (s, 9H), 0.89 (s, 9H), 0.12 (s, 6H); ^{13}C NMR (150 MHz, $CDCl_3$) δ 169.77, 156.49, 143.99, 143.16, 141.39, 127.86, 127.23, 125.30, 120.12, 81.61, 69.28, 67.47, 47.20, 40.05, 28.19, 25.81, 18.02, –4.59, –5.00; HRMS (ESI) *m/z*: $[M+H]^+$ calcd for $C_{30}H_{42}NO_7Si^+$ 556.2725, found 556.2726.

Procedures for the solid-phase peptide synthesis (SPPS).

Step 1: To the solution of compound **6–11** (3 equiv) were added DIC (3 equiv, 0.50 M in NMP) and Oxyma (3 equiv, 0.50 M in DMF). After approximately 2–3 min of preactivation, the mixture was injected to the reaction vessel. The resulting mixture was stirred at 40 °C for 20 min, under microwave irradiation.

Step 2: The resin in the reaction vessel was washed with DMF (× 3) and CH_2Cl_2 (× 3).

Step 3: Fmoc group of the solid supported peptide was removed with 20% piperidine/DMF solution (8 min, room temperature).

Step 4: The resin in the reaction vessel was washed with DMF (× 3) and CH_2Cl_2 (× 3).

Amino acids were condensed onto the solid support by repeating steps 1–4.

2-Chlorotrityl resin (41.5 mg, 0.0647 mmol) in Biotage PP-reactor 2 mL with PTFE frit was swollen with CH_2Cl_2, and then excess solvent was removed by filtration. To the resin were added a solution of Fmoc-D-Trp-OH (**6**, 27.8 mg, 0.0528 mmol) and iPr_2NEt (26 μL, 0.15 mmol) in CH_2Cl_2 (0.23 mL), and stirred for 2 h. The reaction mixture was filtered, washed with DMF (× 3), CH_2Cl_2 (× 3), (CH_2Cl_2/MeOH/iPr_2NEt = 17:2:1) (× 3), DMF (× 3), CH_2Cl_2 (× 3) to afford compound **28**.

The resin-bound D-Trp **28** was subjected to the SPPS protocol (steps 3 and 4), and then 5 cycles of the SPPS

protocol to afford resin-bound peptide **5**.

To resin-bound peptide **5** was added TFA/CH_2Cl_2 1:99 (0.5 mL). After being stirred for 30 min, the reaction mixture was filtered. This procedure was repeated twice. The filtrate was concentrated in vacuo and azeotroped with toluene (× 3) to afford crude peptide **30**, which was used in the next reaction without further purification.

To a solution of peptide **30** in CH_2Cl_2/DMF 9:1 (40 mL) were added 2,4,6-collidine (53 μL, 0.499 mmol), HOAt (29.95 mg, 0.220 mmol), and PyBOP (106 mg, 0.204 mmol) at room temperature. After being stirred overnight, the reaction mixture was concentrated in vacuo, and the residue was diluted with EtOAc and saturated aqueous NH_4Cl. The mixture was extracted with EtOAc (three times). The combined organic layer was washed with brine, dried over $MgSO_4$, filtered, and concentrated in vacuo. To the residue was added a mixture of TFA/iPr_3SiH/H_2O 95:2.5:2.5 (2 mL). After being stirred for 3 h, the reaction mixture was diluted with Et_2O (48 mL), centrifuged at 3500*g* for 5 min at 4 °C, and the Et_2O layer was removed by decantation. This procedure was repeated twice. The obtained crude **1** was purified by reversed-phase HPLC (PEGASIL ODS SP100 ø 20 × 250 mm, MeCN/H_2O/TFA 35:65:0.05, 19 mL/min, room temperature) to afford peptide **1** (13.3 mg, 36% for 15 steps) as a light orange powder: $[\alpha]_D^{20}$ +8.3 (*c* 0.1, MeOH); ^{1}H NMR (600 MHz, DMSO-d_6) δ 10.8 (s, 1H), 8.55 (d, 1H, *J* = 5.5 Hz), 8.16 (d, 1H, *J* = 7.6 Hz), 7.99 (d, 1H, *J* = 6.2 Hz), 7.95 (d, 1H, *J* = 5.5 Hz), 7.70 (br, 1H), 7.68 (br, 1H), 7.50 (d, 1H, *J* = 7.6 Hz), 7.29 (d, 1H, *J* = 8.3 Hz), 7.16 (s, 1H), 7.04 (t, 1H, *J* = 7.6 Hz), 6.97 (t, 1H, *J* = 7.6 Hz), 4.39 (dt, 1H, *J* = 4.8 Hz, 9.6 Hz), 4.28 (m, 1H), 4.25 (m, 1H), 4.18 (t, 1H, *J* = 6.2 Hz), 4.02 (t, 1H, *J* = 7.6 Hz), 3.99 (br, 1H), 3.73 (dd, 1H, *J* = 4.1 Hz, 22.4 Hz), 3.66 (dd, 1H, *J* = 4.1 Hz, 17.2 Hz), 3.26 (dd, 1H, *J* = 4.1 Hz, 14.5 Hz), 2.90 (dd, 1H, *J* = 9.6 Hz, 14.1 Hz), 2.79 (br, 2H), 2.18 (m, 1H), 2.00 (m, 1H), 1.98 (m, 1H), 1.62 (m, 2H), 1.50 (m, 2H), 1.48 (br, 1H), 1.47 (br, 2H), 1.28 (m, 1H), 1.16 (m, 1H), 1.11 (m, 1H), 0.86 (t, 6H, *J* = 6.2 Hz), 0.83 (d, 6H, *J* = 6.2 Hz); ^{13}C NMR (150 MHz, DMSO-d_6) δ 172.51, 171.27, 171.12, 170.37, 170.16, 168.32, 136.23, 126.94, 123.87, 121.02, 118.42, 118.09, 111.52, 110.01, 67.13, 58.84, 58.00, 54.04, 53.27, 52.31, 42.60, 38.64, 38.00, 31.36, 31.09, 29.37, 27.48, 26.82, 23.55, 23.17, 22.55, 22.48, 19.51; HRMS (ESI) *m/z*: $[M+H]^+$ calcd for $C_{36}H_{55}N_8O_9^+$ 743.4087, found 743.4087.

NMR spectra of new compounds

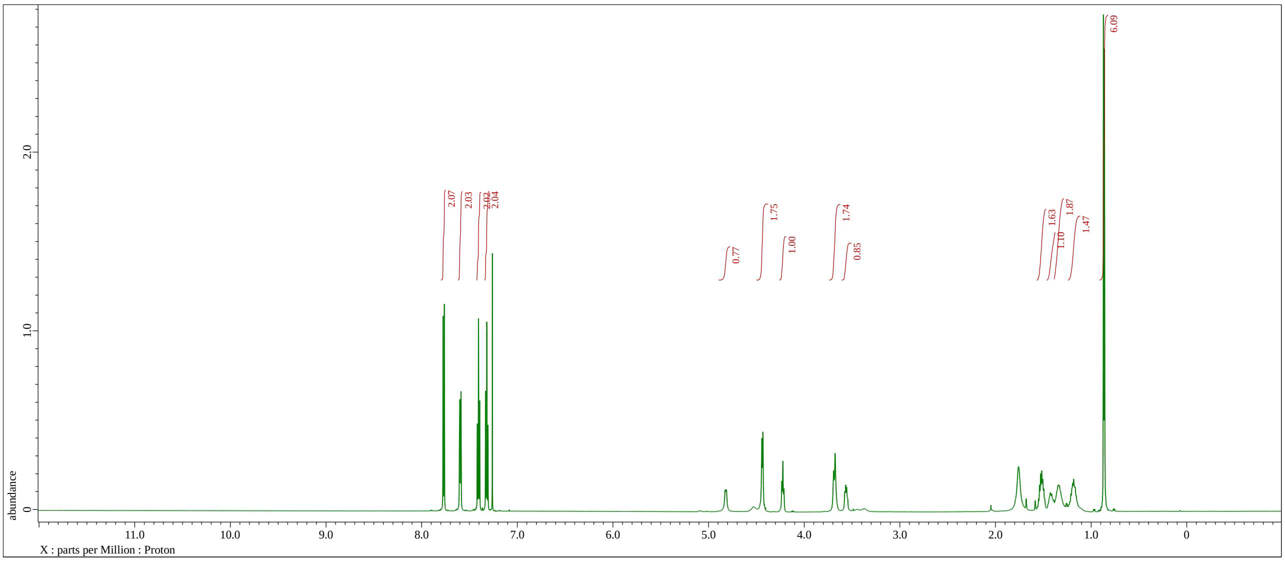

Figure S1: ^{1}H NMR spectrum of **18** in $CDCl_3$ (600 MHz).

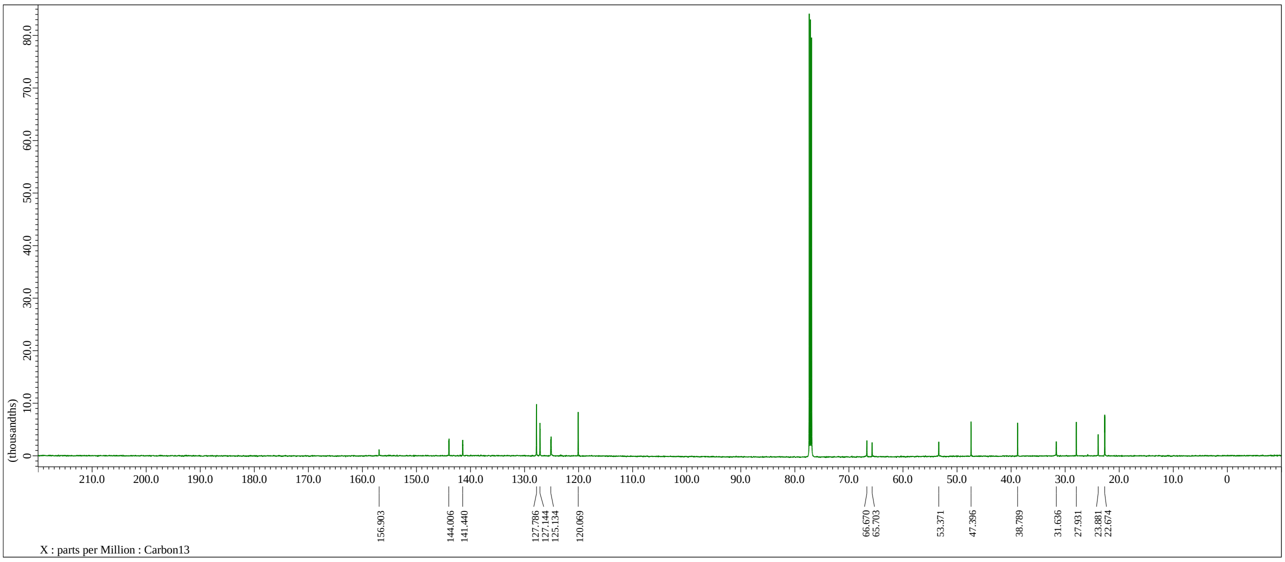

Figure S2: ^{13}C NMR spectrum of **18** in $CDCl_3$ (150 MHz).

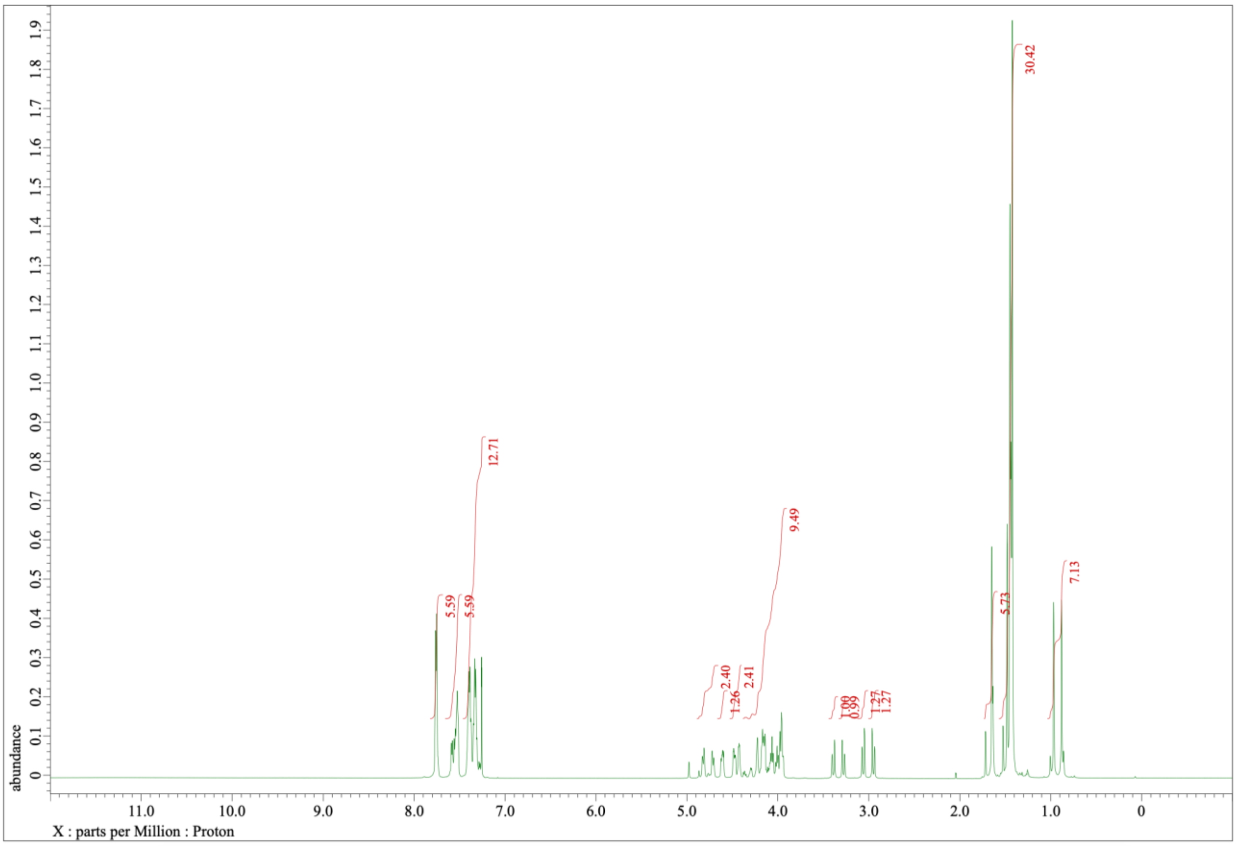

Figure S3: ^{1}H NMR spectrum of **23** (mixture of the rotamer) in $CDCl_3$ (600 MHz).

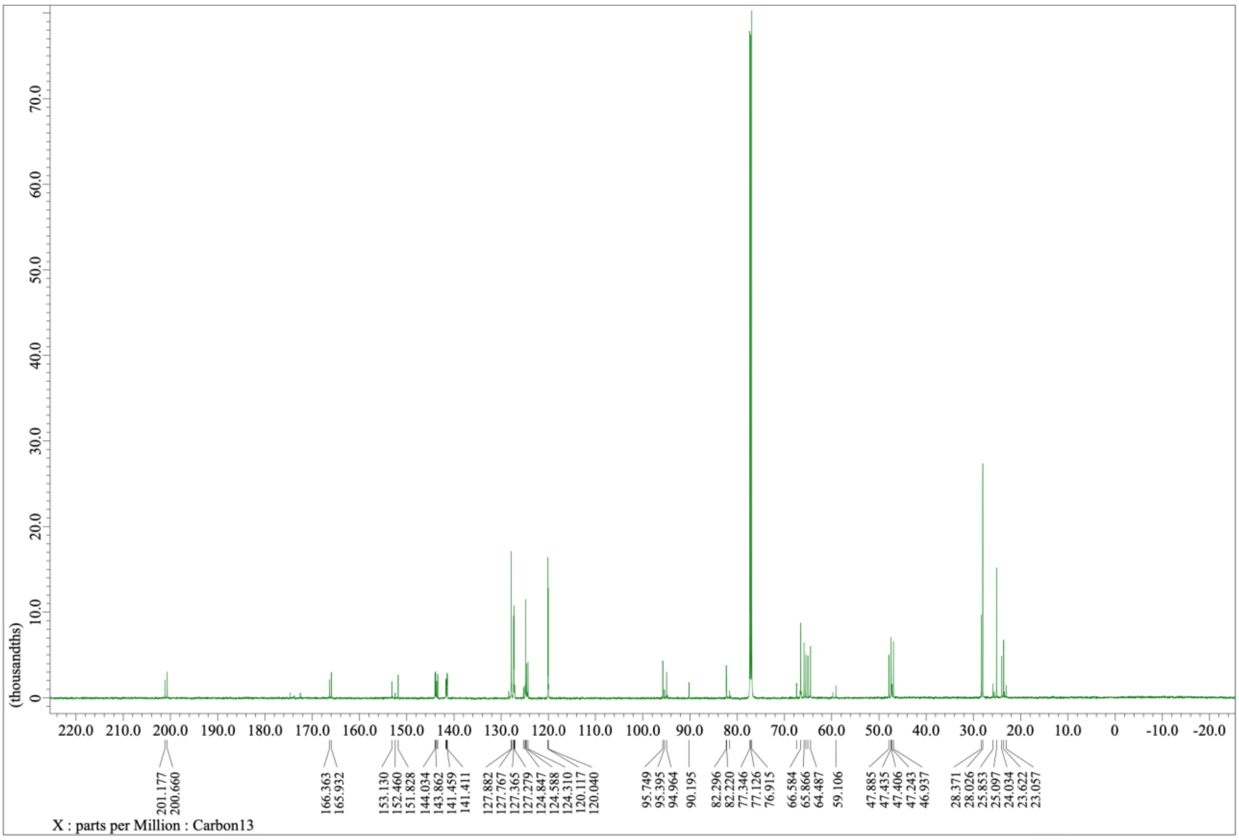

Figure S4: ^{13}C NMR spectrum of **23** (mixture of the rotamer) in $CDCl_3$ (150 MHz).

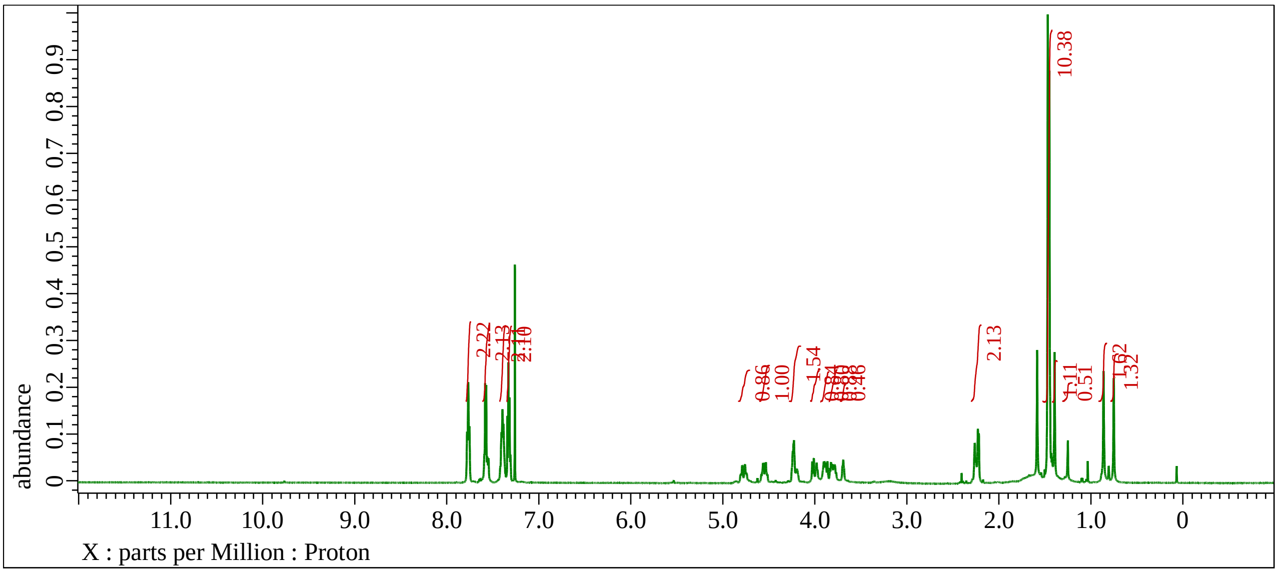

Figure S5: ^{1}H NMR spectrum of **24** in $CDCl_3$ (600 MHz).

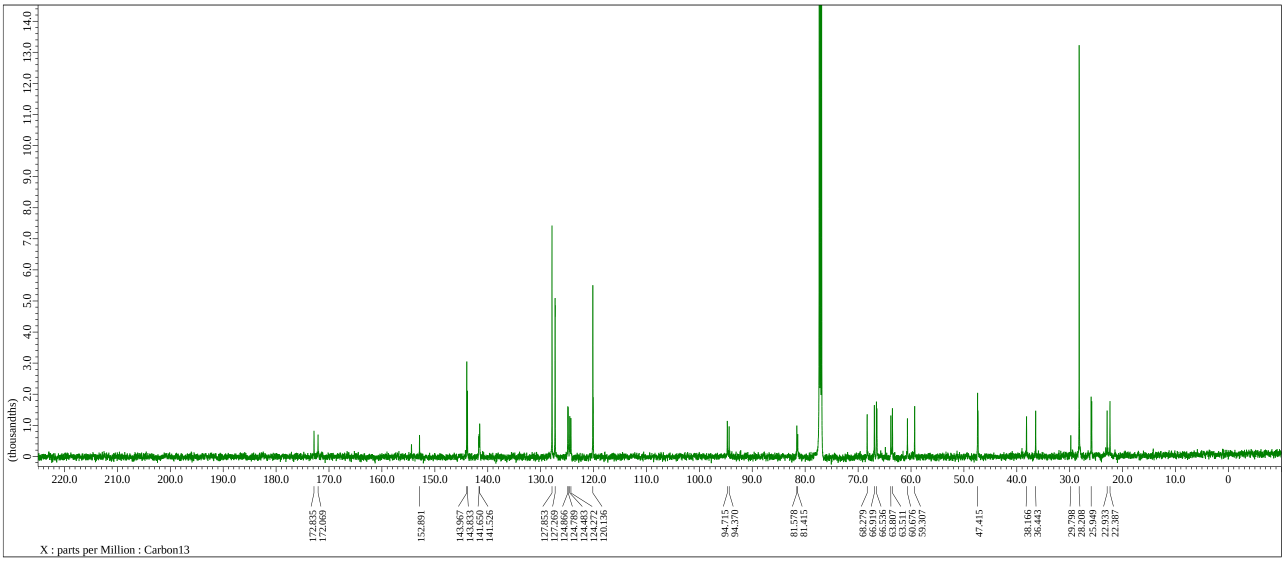

Figure S6: ^{13}C NMR spectrum of **24** in $CDCl_3$ (150 MHz).

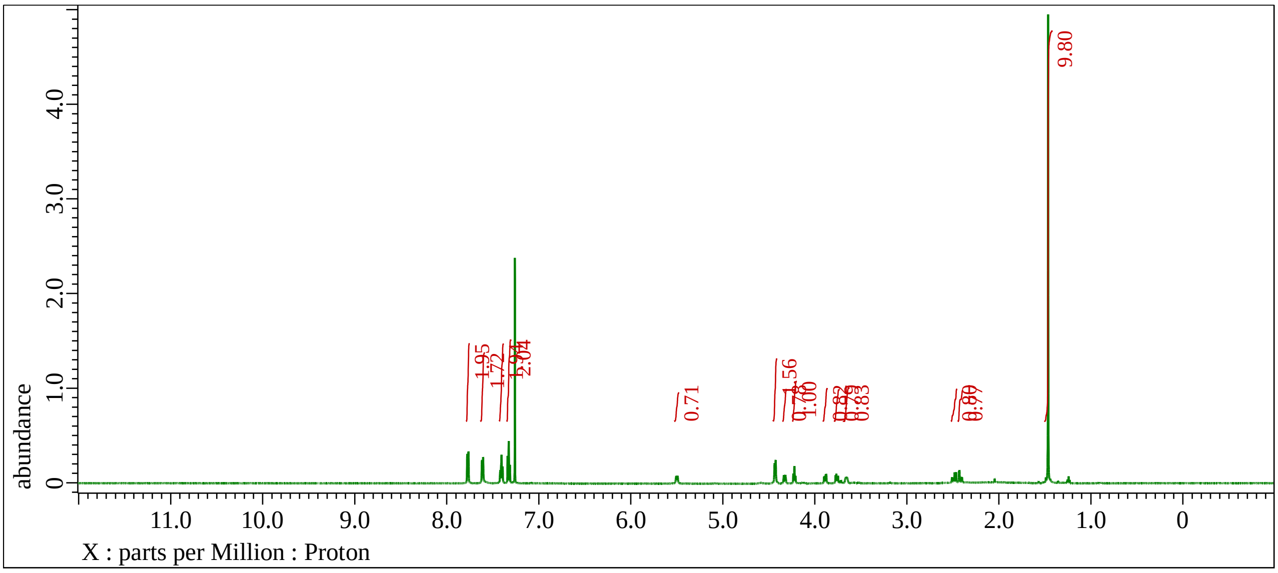

Figure S7: ^{1}H NMR spectrum of **25** in $CDCl_3$ (600 MHz).

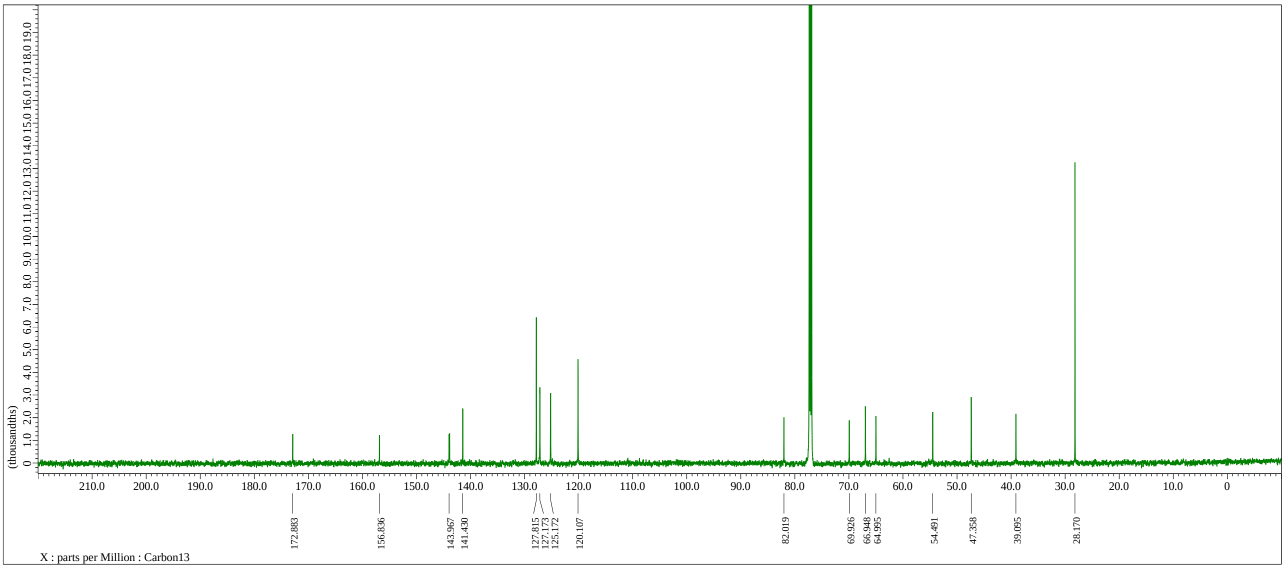

Figure S8: ^{13}C NMR spectrum of **25** in $CDCl_3$ (150 MHz).

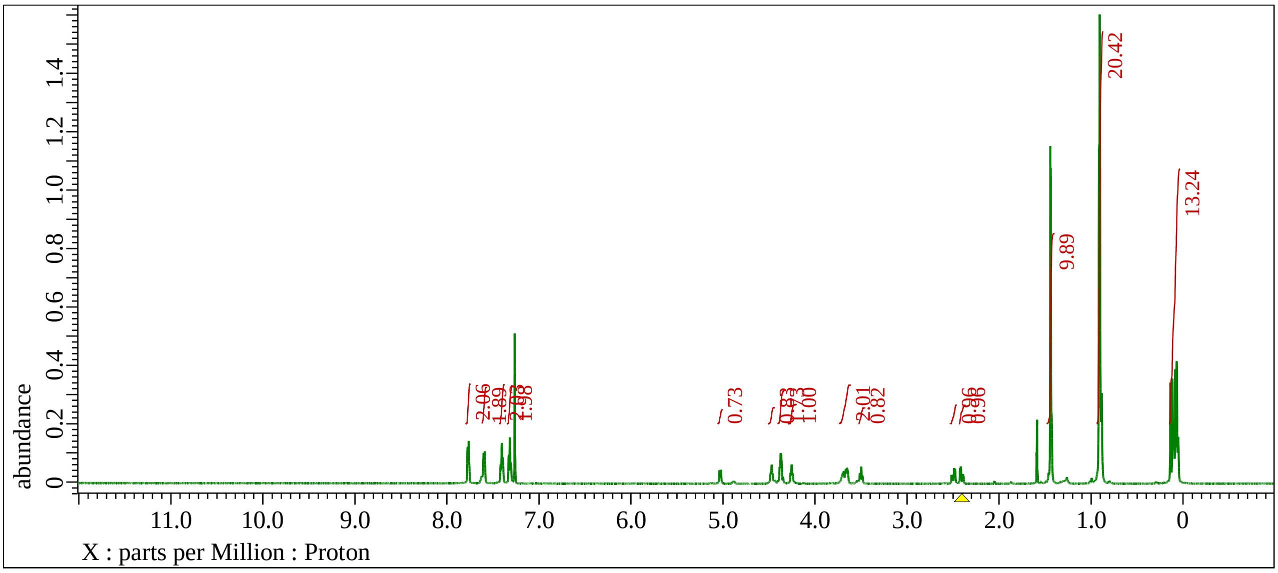

Figure S9: ^{1}H NMR spectrum of **26** in $CDCl_3$ (600 MHz).

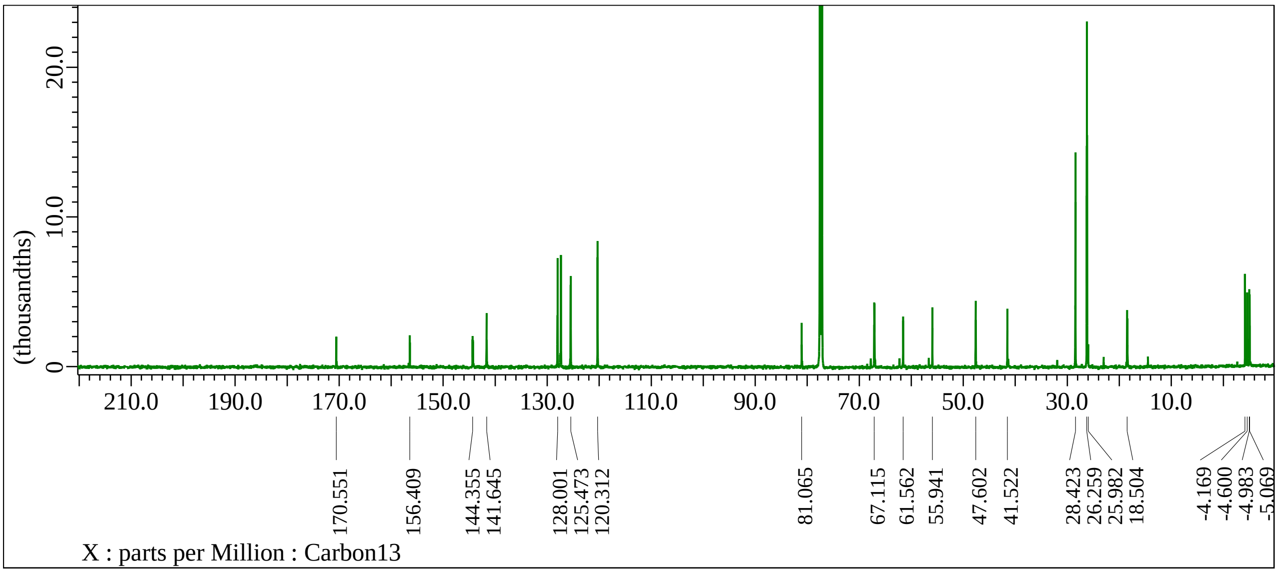

Figure S10: ^{13}C NMR spectrum of **26** in $CDCl_3$ (150 MHz).

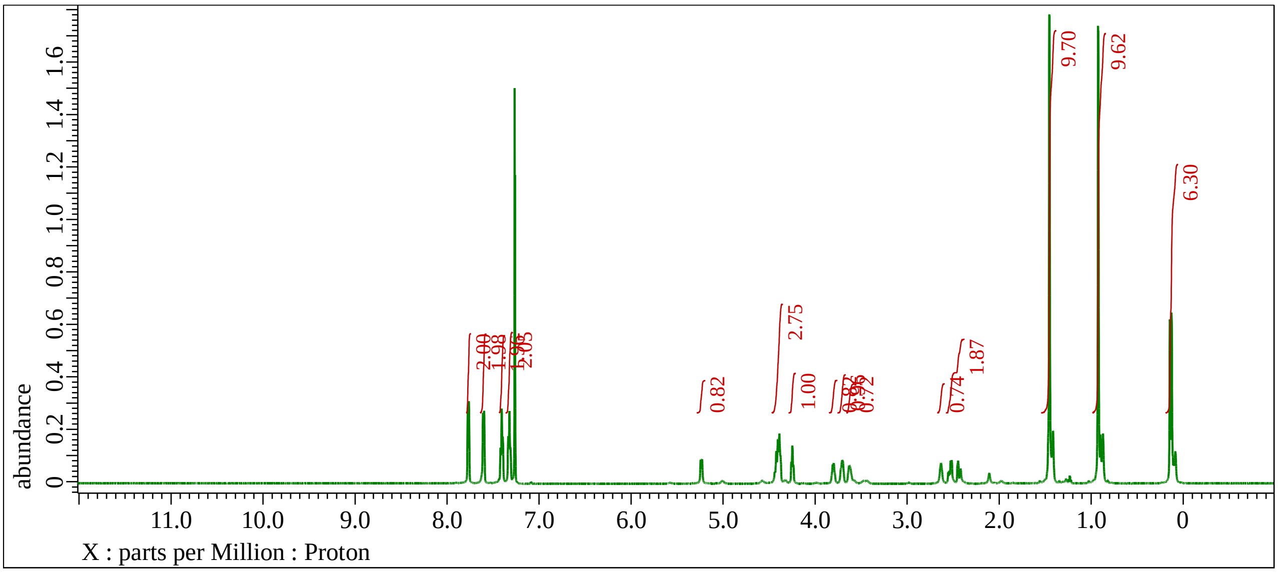

Figure S11: ^{1}H NMR spectrum of **27** in $CDCl_3$ (600 MHz).

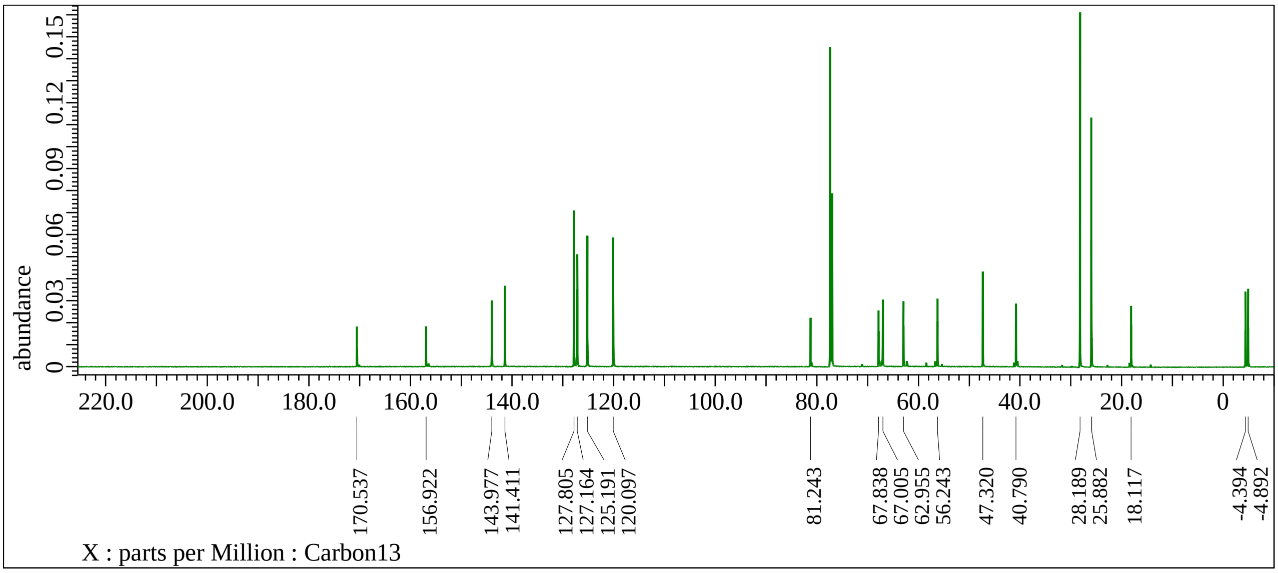

Figure S12: ^{13}C NMR spectrum of **27** in $CDCl_3$ (150 MHz).

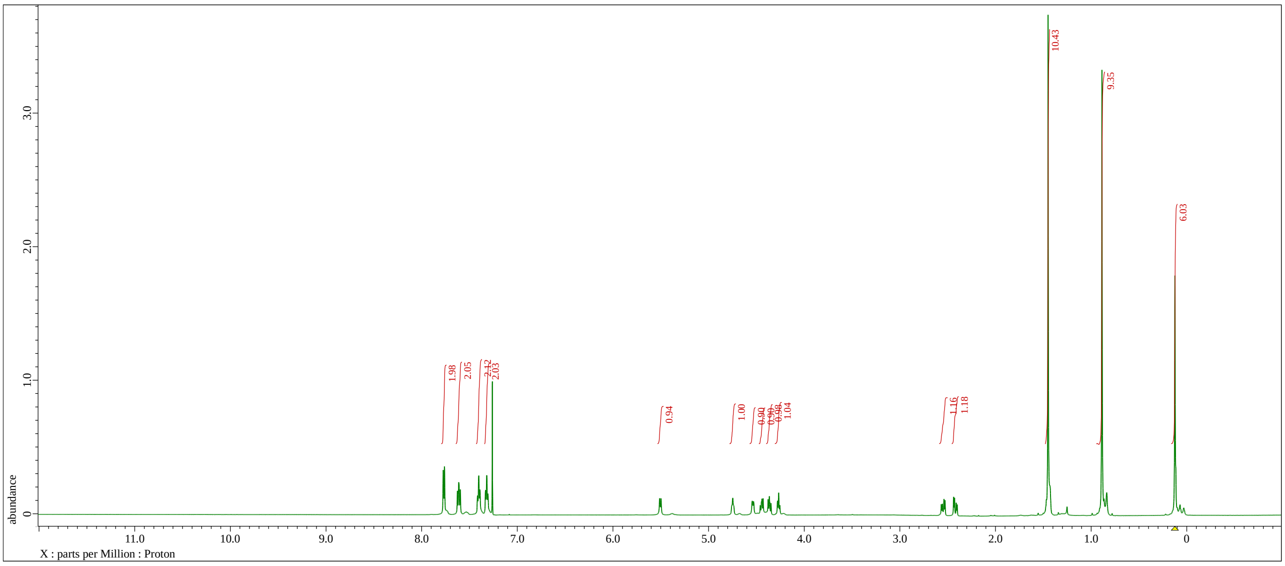

Figure S13: ^{1}H NMR spectrum of **7** in $CDCl_3$ (600 MHz).

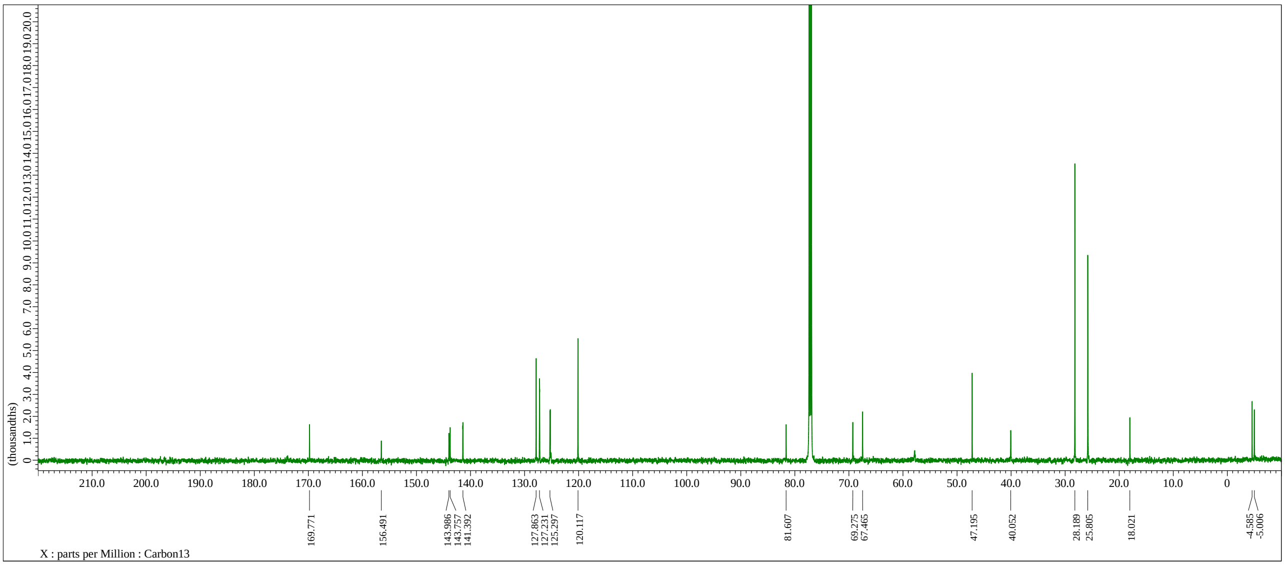

Figure S14: ^{13}C NMR spectrum of **7** in $CDCl_3$ (150 MHz).

NMR spectra of **1**

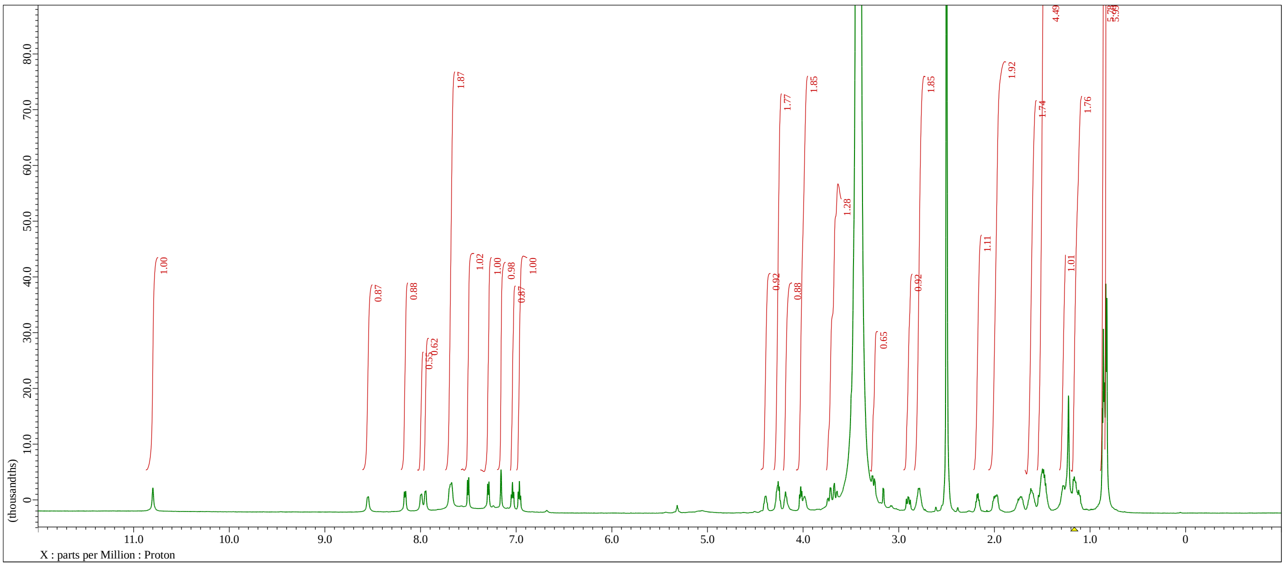

Figure S15: ^{1}H NMR spectrum of **1** in DMSO-d_6 (600 MHz).

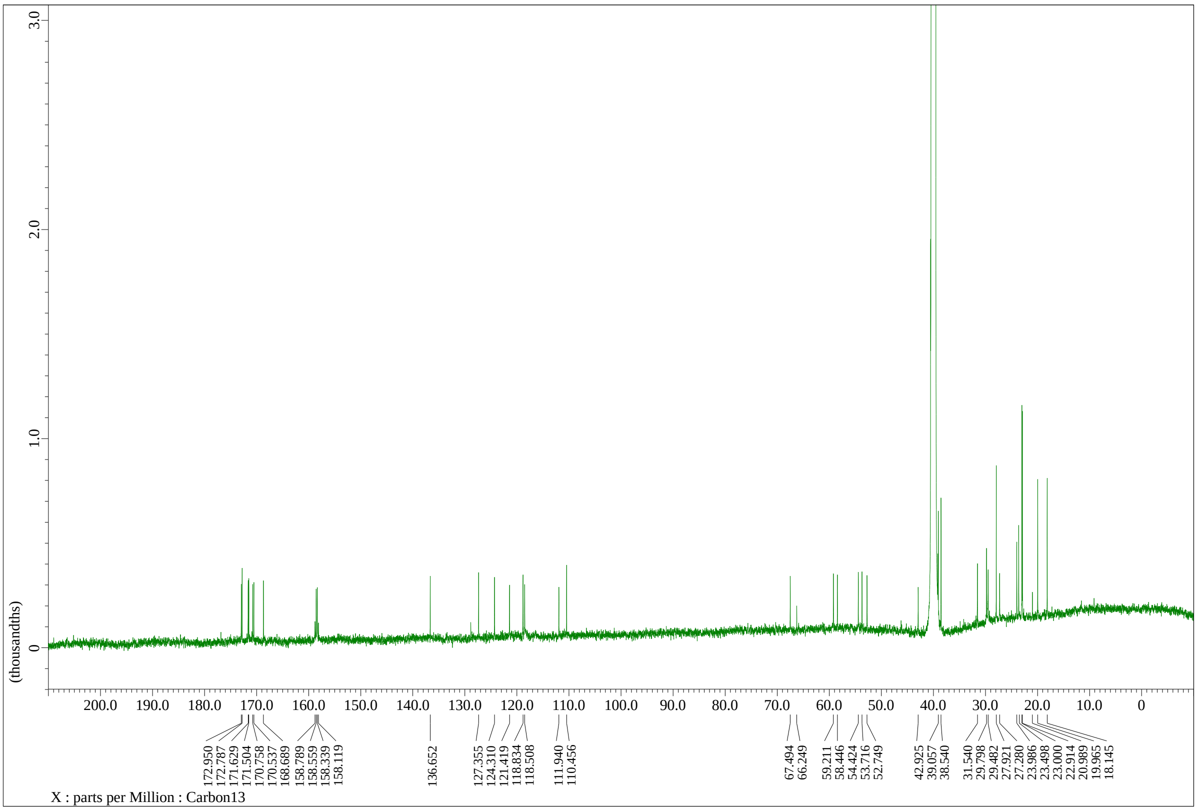

Figure S16: ^{13}C NMR spectrum of **1** in DMSO-d_6 (150 MHz).

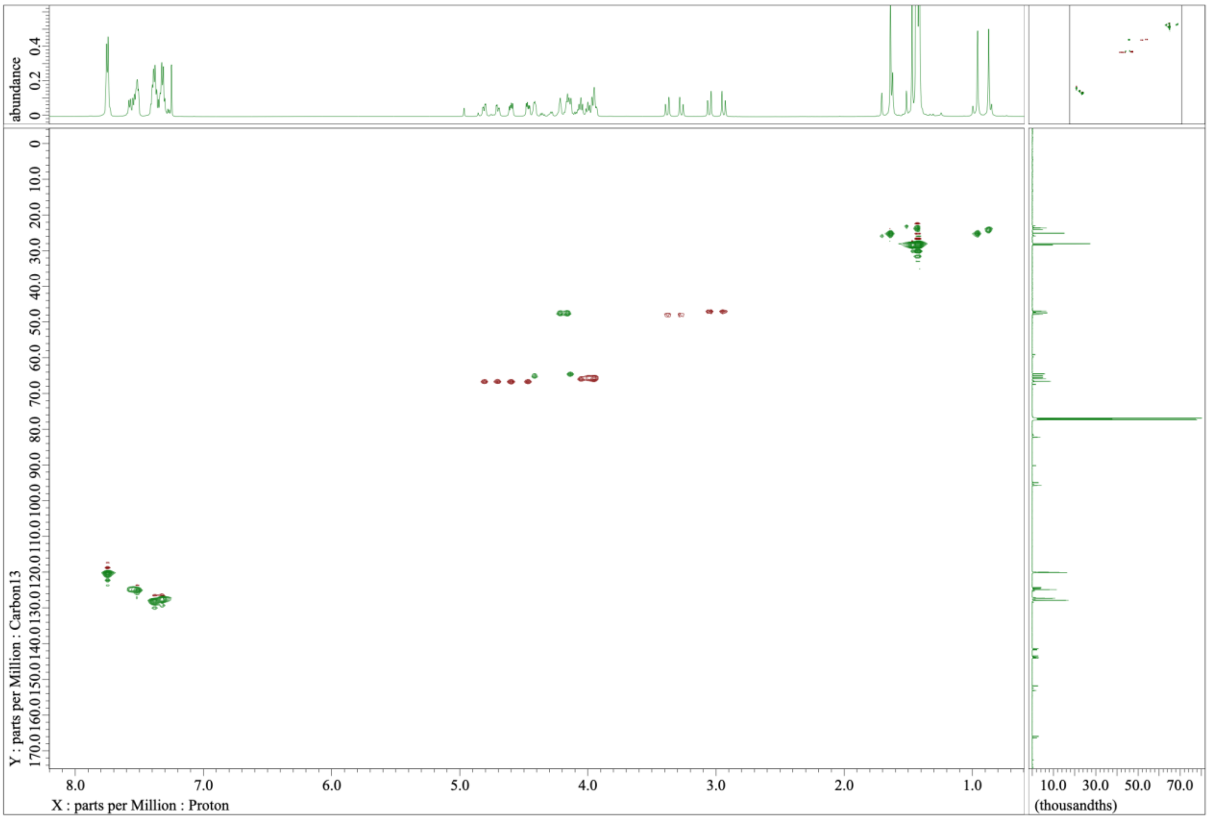

Figure S17: HSQC spectrum of **23** (mixture of the rotamer) in $CDCl_3$ (150 MHz).

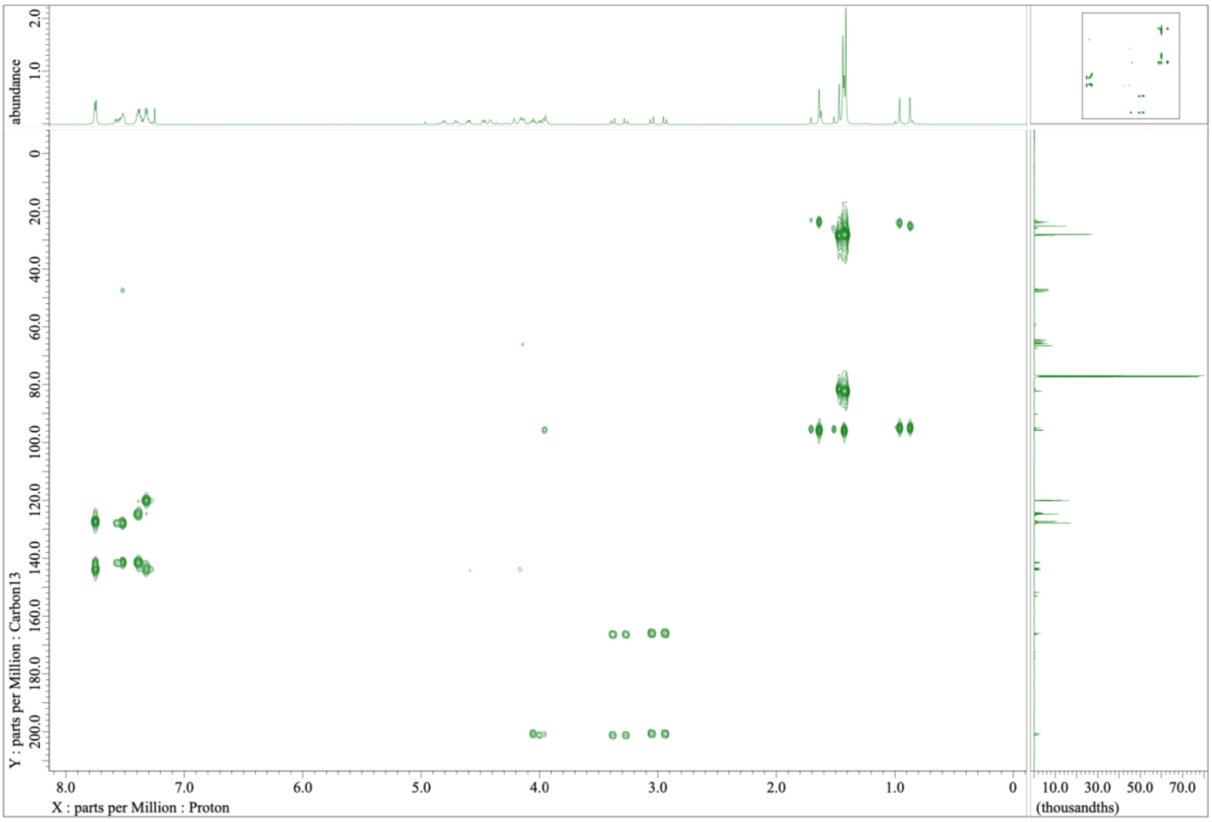

Figure S18: HMBC spectrum of **23** (mixture of the rotamer) in $CDCl_3$ (150 MHz).
